# Opportunities and challenges in the management of celiac disease in Asia

**DOI:** 10.1002/jgh3.12381

**Published:** 2020-07-11

**Authors:** Ashish Agarwal, Ashish Chauhan, Vineet Ahuja, Govind K Makharia

**Affiliations:** ^1^ Department of Gastroenterology and Human Nutrition All India Institute of Medical Sciences New Delhi India

**Keywords:** challenges, diagnosis, epidemiology, intestine

## Abstract

Although once considered uncommon, there is increasing recognition of celiac disease (CeD) in Asia. It is now clear that CeD is a disorder as frequent in certain Asian countries as that in western countries, although it often remains undiagnosed. With increasing awareness and diagnosis, the absolute numbers of celiac patients are expected to increase markedly in Asia. Asia, with 60% of the population of the world, is probably the major “reservoir” of undiagnosed CeD in the world. As Asia has a huge landscape along with highly heterogenous genetic, social, cultural, and nutritional practices, similar heterogeneity is seen in the epidemiology, diagnostic, and therapeutic facilities for CeD in Asia. In this article, we have reviewed the changes in the epidemiology, diagnostics, and management of CeD in Asia and summarized the challenges and opportunities for its emergence in Asia.

## Graphical abstract

Although well recognized in the West, celiac disease is still believed to be uncommon in many Asian countries, except for India. Systematic population‐based prevalence studies have suggested that celiac disease is as frequent in India as that in western countries. A few reports from other Asian countries such as Pakistan, China, Japan, Malaysia, Turkey also support the existence of celiac disease in these countries. Asia is probably the major reservoir of undiagnosed celiac disease in the world.

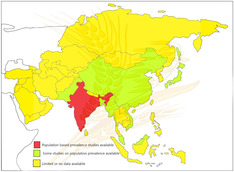

## Introduction

Celiac disease (CeD), once considered to be an uncommon disease affecting mainly children and limited to the western Europe, has now become a global disease.^1^ Such a change in the epidemiology of CeD has occurred due to many advances, including the advent of celiac‐specific serological tests, simplified diagnostic criteria, and—most importantly—increase in the awareness about CeD among physicians.^1^, ^2^, ^3^ While CeD is now well recognized in Europe, North America, South America, and Australasia, it is still believed to be uncommon in many Asian countries, except for countries such as India, Pakistan, Turkey, and certain Middle East countries.^4^, ^5^, ^6^, ^7^, ^8^, ^9^, ^10^ In a joint World Gastroenterology Organization and the Asian Pacific Association of Gastroenterology Working Group position paper in 2015, we have highlighted some of the pertinent challenges about the diagnosis and management of CeD in Asia.^11^ Since then, several advances have occurred in Asia, including new data on the epidemiology, diagnostics, and management strategies of CeD. In this perspective article, we have highlighted some of these advances in the background of existing challenges for CeD in Asia.

### *Changing epidemiology of* *CeD* *in Asia*

We recently conducted a systematic review and meta‐analysis, including 275 818 participants, to estimate the global seroprevalence of biopsy‐confirmed CeD, and we observed that the global seroprevalence and prevalence of biopsy‐confirmed CeD was 1.4% (95% confidence interval [CI], 1.1–1.7%) and 0.7% (95% CI, 0.5–0.9%), respectively.^12^ These estimates suggest that there are approximately 40–60 million patients with biopsy‐confirmed CeD globally. Furthermore, the pooled prevalence of CeD has increased from 0.6% (95% CI, 0.5–0.7%) in 1991 to 2000 to 0.8% (95% CI, 0.5–1%) between January 2011 and March 2016, suggesting an increase in the prevalence of CeD over time.^12^

Not only the prevalence but incidence has also been rising throughout the Western world. The pooled average annual incidence of CeD has been rising by 7.5% (95% CI, 5.8, 9.3) per year over the past several decades. The pooled incidence of CeD in women and men has been reported to be 17.4 (95% CI, 13.7, 21.1) and 7.8 (95% CI, 6.3, 9.2) per 100 000 person‐years, respectively.^13^

While CeD has now been well recognized in many Southeast Asian countries, including India and Pakistan, and Middle East countries such as Iran, Saudi Arabia, and Turkey, there have been some very interesting reports that have emerged from India, China, Malaysia, Japan, and Vietnam since our last review, published in 2015.^11^ In a systematic review and meta‐analysis, we showed that the pooled seroprevalence (positive serological tests) of CeD in Asia was 1.6% in 47 783 individuals, and pooled prevalence of biopsy‐confirmed CeD in Asia was 0.5% in 45 955 individuals.^12^

In the northern part of India, where wheat is a predominantly consumed cereal, we had observed the prevalence of biopsy‐confirmed CeD to be 1.04% (1 in 96) and seroprevalence to be 1.44% (1 in 69) in a population‐based study including 2879 healthy adults.^8^ Furthermore, a large population‐based study recruiting 23 331 healthy adults from three different regions of India showed a high age‐adjusted prevalence of CeD of 0.67%.^9^ Interestingly, there was a difference in the prevalence between the three geographical regions of India, with the highest being in the northern part of India (1.23%), the lowest in the southern part of India (0.10%), and in‐between in the northeastern India (0.87%). While people in all the three regions had equal genetic susceptibility as determined by the population prevalence of HLA‐DQ2 and HLA‐DQ8, the differences in the prevalence were likely because of the differences in the wheat‐eating pattern, with the highest being in the northern part of India (455 g/day) and lowest in the southern part of India (25 g).^9^ Based on the above‐mentioned population studies, while we expect that 5–8 million Indians may have CeD, only a fraction has been diagnosed so far.

A population‐based prevalence study including 19 778 healthy asymptomatic young Chinese individuals from 27 geographic regions suggested a prevalence of CeD of 0.76% in Shandong province, where wheat is the staple diet.^14^ In another recent study including 2277 patients with gastrointestinal symptoms in four major ethnic groups of Xinjiang Uyghur Autonomous Region, China (1391 Han, 608 Uyghur,146 Kazakh, and 132 Hui), the seroprevalence and prevalence of biopsy‐confirmed CeD was found to be 1.27% (95% CI, 0.81–1.73%) and 0.35% (95% CI, 0.11–0.59%), respectively.^15^ The frequency of the HLA‐DQ2 and/or DQ8 haplotype was the highest in the Uyghur (52.1%), followed by the Hui (44.4%), the Kazakh (40.0%), and the Han (39.4%). CeD was found to be three times more common in rural parts with significantly higher wheat consumption compared to urban living subjects. Yet another systematic review and meta‐analysis by Yuan *et al*. has predicted that CeD is not uncommon in China.^16^

Although little is known about CeD prevalence in Russia, scattered reports have suggested a prevalence varying from 0.20% to 0.57% in their general population.^17^ In a study from Malaysia, the seroprevalence of CeD was found to be 1.25% (95% CI, 0.78–1.72%) in a relatively small study including 562 young healthy volunteers.^18^, ^19^ Although there are suggestions of existence of CeD in Southeast Asia based on the reports from Japan,^19^ Singapore,^20^ and Vietnam,^21^ the prevalence in these areas is either low or unknown. Furthermore, there are no formal reports on CeD from Taiwan, Indonesia, Korea, and many other Asian countries.

The two most important reasons for the variations in the prevalence of CeD in Asia are dietary practices and genetic diversities (HLA and non‐HLA genes). As is clear from the above‐mentioned studies, the prevalence of CeD follows the dietary behavior, with higher prevalence reported in regions where wheat (gluten‐containing cereal) is the staple food (e.g. North India and northern China). Although rice has been the staple cereal in many Asian countries, there has been a change in dietary practices, with widespread diffusion of western dietary habits leading to increasing consumption of gluten‐containing cereals, thus leading to an increased prevalence of CeD in Asian countries as well. We have recently reviewed these two factors in Asia.^22^

## Identification of challenges and suggested strategies

### *Steps toward increasing awareness about the recognition of* *CeD* *in Asia*

#### *Establish the burden of disease in Asia*

We need to first establish the population prevalence of CeD across the region. While population‐based studies are ideal to estimate the prevalence CeD in any particular country/region, they are labor intensive and expensive. An alternative approach may be assessing the prevalence of CeD in high‐risk conditions where prevalence of CeD has been shown to be many folds higher than that in the general population, such as type I diabetes mellitus, irritable bowel syndrome, anemia, chronic diarrhea, or short stature. These studies can be performed in the hospital setting and may not involve much cost. If the results of these studies indicate the existence of CeD, population‐based studies can then be undertaken to assess the true prevalence in these regions. A multinational study to assess the prevalence of CeD in high‐risk group patients in Asia has already been initiated by the author.

#### *Increasing awareness about wide clinical spectrum of* *CeD*

While traditionally believed that the hypersensitivity to gluten peptides is limited to the small intestine in patients with CeD, it is now known that CeD affects many other organs, including skin, liver, kidney, bone, and brain, and hence, CeD is now considered to be a systemic disorder altogether.^23^, ^24^, ^25^, ^26^ While symptoms of CeD were typically defined as those related to malabsorption, such as chronic diarrhea, steatorrhea, weight loss, failure‐to‐thrive in children, short stature, irritability, excessive flatulence, and recurrent aphthous ulcers (*classical CeD*),^27^, ^28^, ^29^ it is increasingly being recognized that the patients can have extraintestinal manifestations in the absence of or minimal gastrointestinal symptoms such as short stature, ataxia, hypertransaminasemia, cirrhosis of liver, and osteomalacia (*atypical CeD*).^30^ Education and increased awareness of medical communities across specialties, as well as during initial years of training, is thus needed to allow for a timely diagnosis of CeD and institution of early intervention, which will prevent organ damage. We also need to target the primary care physicians and family physicians as they generally form the first contact for patients. Gastroenterologists in Asian countries can play a key role in educating them and thus empowering them to play a pivotal role in increased detection of CeD.

### *Setting up of facility for diagnosis of* *CeD*

#### *Serological testing*

Celiac‐specific serological tests are the center stage of both the screening of the suspected patient and in the diagnosis of CeD. The serological tests commonly used include IgA subclass of antitissue transglutaminase (anti‐tTG) antibody, antiendomysial antibody (EMA), and antideamidated gliadin peptide (DGP) antibody. A recent systematic review including 56 original studies and 12 previous systematic reviews reported strong evidence regarding the high accuracy of IgA anti‐tTG assay for the diagnosis of CeD with pooled sensitivity of 92.8% (95% CI, 90.3–94.8%) and pooled specificity of 97.9% (95% CI, 96.4–98.8%).^31^ Anti‐EMA IgA testing was associated with a lower sensitivity of 73% (95% CI, 61.0–83.0%) but had a high specificity 99.0% (95% CI, 98.0–99.0%).

Currently, most of the celiac‐specific serological test kits are imported from Europe and North America. These tests have their diagnostic accuracy evaluated for Caucasian populations, and thus, the cut‐offs of the antibody level are determined for these populations. With a difference in the genetic makeup and the amount of gluten ingestion, these cut‐offs for a positive test, which have been determined for the Caucasian population, may not have similar diagnostic accuracy for Asian patients. We have observed a high intra‐assay variation, as well as variation between two racially distinct geographical populations in the diagnostic performance of IgA anti‐tTG assays for the diagnosis of CeD.^32^ The assay with the highest sensitivity in the Indian population had the lowest sensitivity in the Canadian population. This highlights the importance of the assessment of performance of a particular assay in the specific population being tested to avoid both over‐ and underdiagnosis of CeD.

Point‐of‐care tests, wherein blood/serum along with buffer solution is placed on a test strip, which diffuses down a strip with a positive test reflected by a solid line in the test window, are being increasingly used for diagnosis of CeD. They have high diagnostic accuracy (sensitivity and specificity around 94%) and are rapid and easy to perform, with no requirement of laboratory or experienced staff, and have the potential to increase recognition of CeD, facilitate early diagnosis, and reduce costs.^33^

#### *Demonstration of villous abnormalities*

Histological examination of intestinal mucosa for the demonstration of villous abnormalities is one of the gold standards for the diagnosis of CeD. A correct assessment of duodenal mucosal specimen needs multiple biopsies from the second part of duodenum and at least one biopsy from the duodenal bulb as per the current recommendation.^2^, ^34^, ^35^, ^36^ Furthermore, the biopsies need to be oriented properly before mounting them on a paraffin block. The modified Marsh classification used for grading of the severity of mucosal changes in small intestinal biopsies is descriptive and qualitative, and accuracy of reporting depends on the experience and judgment of the pathologist.^37^ As this grading is based on visual impression of the reading pathologist, there is a high degree of interobserver and intraobserver variation in the assessment of the severity of villous abnormalities and, thus, poor reproducibility.^38^, ^39^ There has been an increasing interest in quantitative histological reporting, where the crypt depth, villous height, and number of intraepithelial lymphocytes per 100 epithelial cells are measured using a microscope with a calibrated micrometer.^37^, ^38^, ^40^ We have recently reported a qualitative assessment of villous abnormalities using a computer‐based quantitative assessment.^41^ There is a need to increase awareness and training of the pathologists to adapt to the new system, which consumes more time but has higher objectivity, interobserver agreement, and reproducibility.^38^ There is a need to explore artificial intelligence and deep machine learning in the assessment of duodenal mucosal biopsies.

### *Variation in the diagnostic criteria*

There are four pillars in the diagnosis of CeD, including clinical symptoms, positive celiac‐specific serological test, presence of villous abnormalities, and the response to gluten‐free diet. The diagnostic criteria of CeD varies between that recommended for children and the adults. While most national and international societies recommend duodenal biopsies in addition to the celiac‐specific serological tests, the most popular diagnostic criteria for children developed by the European Society of Gastroenterology Hepatology and Nutrition (ESPGHAN) has provided the option of a nonbiopsy approach for those children in whom the anti‐tTG antibody is more than 10‐fold higher.^2^ The basis of a nonbiopsy approach is based on the evidence of high predictive value of the presence of villous abnormalities if the anti‐tTG value is more than 10 times the cut‐off value for a positive test.^42^, ^43^, ^44^ While serology‐based diagnosis is a welcome state, this approach is often misused by physicians, and a diagnosis of CeD is made even at low‐titer anti‐tTG Ab without duodenal biopsies being performed.^45^ Lack of unified guidelines between different societies and for both children and adults leads to confusion and, at times, questioning of the initial diagnosis of CeD by the patient, as well as the physician.^46^

All efforts should be made to make a definite diagnosis of CeD before starting the treatment, that is, gluten‐free diet, and a therapeutic challenge‐based diagnosis should be avoided. In summary, there is a need to develop Asian diagnostic criteria based on standardized Asian cut‐off values of the serological tests and the description of normative histological crypt‐villous characteristics of Asians.

### *Issues related to management of CeD patients in Asia*

Lifelong adherence to gluten‐free food (GFD) is the cornerstone of the successful treatment of CeD. There are three major difficulties in proper adherence to GFD in Asian countries:

#### *Lack of expert dieticians*

The pillar of treatment of CeD is proper counseling by a dietician trained in the management of CeD. These patients and their families require repeated consultations and monitoring. Therefore, there is a need to train our dieticians in the management of CeD.

#### *Widespread availability of reliable gluten‐free diet in food supply chain*

Because of its viscoelastic properties, gluten is used extensively in the food industry, and thus, naturally occurring gluten‐free food products such as chips or wafers may contain gluten.^47^ While CeD is emerging in many Asian countries such as India, there is a lack of large industrial production of gluten‐free food items, and the majority of gluten‐free food product manufacturing units are either small or medium sized. The quality of gluten‐free food products is also not often assured. In a study including 820 gluten‐free food products tested, we observed that 86 (10.5%) had gluten content above the prescribed upper limit of 20 mg/kg (unpublished data). Because of a lack of legislation, packaged food products are not labeled for its gluten content. While gluten‐free products are being made available in supermarkets and through eCommerce platforms, the supply is limited to major cities. There is a need for the supply of gluten‐free food products widely.

### *Defining a strategy for monitoring and long‐term follow up*

While there is an ongoing debate in many western countries regarding the nature and the structure of follow up for patients with CeD and the targets of treatment including mucosal healing, Asia also needs to define the schedule of the follow up for Asian patients with CeD.

### *Creation of a celiac working group in Asia*

A welcome step was when a working group of 13 members from the Asia‐Pacific region and World Gastroenterology Organization reviewed relevant literature on issues specific to the Asia‐Pacific region for the diagnosis and management of CeD.^11^ Recently, the Asia‐Pacific Association for Gastroenterology created a formal working group on CeD to conduct relevant research to unravel the burden of CeD in Asia. Furthermore, a pan‐Asian study (including at least eight Asian countries) has been planned to assess the prevalence of CeD in high‐risk groups as a first step toward estimating the burden of CeD in Asia.

## Conclusion

With two of the most populous countries, India and China, in the world, the absolute numbers of patients with CeD in Asia may exceed the total number of patients in Europe and North America combined together. Asia is probably the major “reservoir” of undiagnosed CeD in the world. Asia has a huge landscape and population, along with highly heterogenous genetic, social cultural, and nutritional practices. Similar heterogeneity is seen in the CeD epidemiology, awareness, and availability of diagnostic and treatment facilities. It is now time for Asian countries to define the extent of disease and start preparing to handle the impending epidemic of CD. Alas, “Rome was not built in a single day,” and there is yet a long way to go.

## Declaration of conflict of interest

We declare no competing interests.
